# Persistent Low Toxoplasma IgG Avidity Is Common in Pregnancy: Experience from Antenatal Testing in Norway

**DOI:** 10.1371/journal.pone.0145519

**Published:** 2015-12-29

**Authors:** Gry Findal, Babill Stray-Pedersen, Ellen K. Holter, Tone Berge, Pål A. Jenum

**Affiliations:** 1 University of Oslo, Institute of Clinical Medicine, Oslo, Norway; 2 Division of Women and Children, Oslo University Hospital, Oslo, Norway; 3 Department of Medical Microbiology, Oslo University Hospital, Oslo, Norway; 4 Department of Medical Microbiology, Vestre Viken Health Trust, Drammen, Norway; Hong Kong Institute for the Humanities and Social Sciences, HONG KONG

## Abstract

The parasite *Toxoplasma gondii* might harm the fetus if a woman is infected during pregnancy. IgG seroconversion and significant increase in IgG antibody amount in pregnancy indicates maternal infection. Presence of toxoplasma immunoglobulin M (IgM), immunoglobulin G (IgG) and low IgG avidity in a single serum sample indicates possible maternal infection, but positive toxoplasma IgM and low IgG avidity may persist for months and even years. We aimed to evaluate avidity development during pregnancy in a retrospective study. Serial blood samples from 176 pregnant women admitted to Oslo University Hospital 1993–2013 for amniocentesis because of suspected toxoplasma infection were included. Data were obtained from journals and laboratory records. The avidity method used was based on Platelia Toxo IgG assay. Mean maternal age at first serology was 29.9 years (SD 5.2, range 18–42). In 37 (21%) women only the avidity increased from low to high in < 3 months. In 139 (79%) the IgG avidity remained below the high threshold ≥ 3 months and within this group 74 (42%) women had stable low IgG avidity during the observation period. Median gestational age at first test was 10.6 weeks (range 4.6–28.7). Fetal infection was detected in four children, but none among children whose mother had stable low IgG avidity. The first antenatal toxoplasma serology should ideally be collected in early pregnancy and if stable values of toxoplasma IgM and low IgG-avidity are detected in a second sample after three to four weeks, the need for amniocentesis can be questioned.

## Introduction

Primary infection with *Toxoplasma gondii* during pregnancy may result in severe damage to the fetus if the parasites are transmitted through the placenta [1]. The risk of transmission and the severity of fetal disease depend on gestational age at the time of maternal infection [2]. The fetus becomes infected during the period of maternal parasitemia, before the development of toxoplasma-specific antibodies [1]. Maternal antibodies protect the foetus, and infection prior to pregnancy does not affect the fetus [3]. Maternal toxoplasma infection is usually asymptomatic; therefore, the diagnosis relies mainly on serologic tests collected through screening programmes or random testing. The presence of toxoplasma immunoglobulin G (IgG) antibodies confirms ongoing or previous infection, and the presence of toxoplasma immunoglobulin M (IgM) antibodies indicates a possible ongoing infection. However, discrimination between past and recent infection is challenging, as an individual can be positive for toxoplasma IgM antibodies for several months or years after primary infection [4–6].

In recent decades, determination of toxoplasma IgG avidity has been included as a standard diagnostic tool to improve the estimation of the time of infection acquisition [7, 8]. The IgG avidity test measures antibody binding force, which is low in the early stage after primary infection but generally increases with time. High toxoplasma IgG avidity indicates that an infection likely occurred at least four months earlier [8, 9]. However, several studies have shown that IgG avidity can remain low for a longer period following infection [6, 8, 9]. This may be a normal reaction after infection in some individuals, due to immunological changes during pregnancy or a response to antibiotic treatment [5, 10]. Therefore, toxoplasma infection is impossible to confirm during pregnancy based solely on low toxoplasma IgG avidity.

The “Prevention of congenital toxoplasmosis in Norway” project, performed two decades ago, recommended the screening of pregnant women for toxoplasma infection [11]. However, health authorities did not find sufficient evidence for implementing a screening programme, mainly because of uncertainty concerning the effect of antenatal treatment [12]. Nevertheless, toxoplasma testing during pregnancy has steadily increased, at least in southern Norway [13, 14]. The first antenatal visit is generally in gestational week 8 to 12, and if requested, toxoplasma serology is commonly performed during this visit. When the test is performed at the end of the first trimester, physicians often face a dilemma: the combination of IgM positivity and low IgG avidity may indicate recent infection and warrant a recommendation for amniocentesis; however, this result does not necessarily indicate that the infection occurred during the last three months.

To better understand the possible impact of a low toxoplasma IgG avidity result in pregnancy, we re-examined the laboratory records of pregnant women who were repeatedly tested for toxoplasma antibodies and who later underwent amniocentesis, with particular focus on low toxoplasma IgG avidity persisting for three months or longer.

## Materials and Methods

### Study population

In this retrospective study, the toxoplasma antibody results of serum samples collected from 01 September 1992 until 31 December 2013 from 352 women who had undergone amniocentesis as part of their prenatal diagnosis at Oslo University Hospital due to suspected primary *T*. *gondii* infection were evaluated for inclusion. The pregnant women were mainly referred from health centres in the Southeast Health Region, which includes more than 50% of Norway’s pregnant population [15]. Only women with initial low toxoplasma IgG avidity and follow-up sera collected and analysed over a period of at least three months were included. A total of 176 women provided 542 serum samples. The women were divided into three groups according to their toxoplasma serology profile: IgG seroconversion, significant IgG antibody increase or IgM positivity and low IgG avidity. These groups were further subdivided into three subgroups: IgG avidity change from low to high, significant increase in IgG avidity below the high threshold, and stable low IgG avidity. The duration of low avidity was calculated from the time of the first sample with a valid IgG avidity value until the final sample with low avidity.

Congenital infection was confirmed by positive toxoplasma DNA detected by polymerase chain reaction (PCR) in amniotic fluid or cord blood, a positive mouse inoculation test, positive toxoplasma-specific IgM, IgA in serum samples collected during the first year of life (with prompt re-examination in cases of positive cord blood due to the possibility of maternal contamination) or persistent positive toxoplasma-specific IgG serum samples during the first year of life [16].

Demographic data, information on toxoplasma serology, follow-up data and birth outcome were collected from patient journals, laboratory records and the mothers’ health cards.

### Serum analyses

All serum samples were analysed at the Norwegian Institute of Public Health (before 2002) and at the Toxoplasma Reference Laboratory at Oslo University Hospital (established in 2002) by indirect enzyme-immunoassay (EIA), (Platelia Toxo IgG and Toxo IgM, Diagnostic Pasteur/Bio-Rad, Marnes la Coquette, France) and Toxo-Screen DA IgG and Toxo-ISAGA IgM (bioMérieux, Marcy l´Etoile, France). Since June 2005, in addition to EIA Toxo IgG and Toxo IgM assays were performed with microparticle enzyme-immunoassay (MEIA) technique (Axsym, Abbott, Wiesenbaden, Germany). Chemiluminescence (CMIA) assay replaced the MEIA, Toxo IgG assay in June 2009 and the Toxo IgM assay in January 2010 (Architect, Abbott, Wiesenbaden, Germany). Until June 2005, the avidity method was performed as previously described using an in-house method based on the Platelia Toxo IgG assay [8]. Thereafter, the commercially available Platelia IgG avidity test was used (Bio-Rad, Marnes la Coquette, France). Until June 2005, the avidity results were expressed as the percentage of antibodies resistant to elution by urea [8] and after June 2005, the results were expressed as an avidity index (AI) and were interpreted according to the manufacturer’s recommendations. Values greater than 20% and AIs greater than 0.5 were considered as high avidity and to be indicative of previous infection, values of 20–15% and AIs of 0.5–0.4 were considered to indicate borderline avidity, and values of less than 15% and AIs less than 0.4 were considered to indicate low avidity and possible recent *T*. *gondii* infection.

A significant antibody increase was defined as a greater than 2x increase in IgG antibody level expressed in IU/ml. A significant increase in IgG avidity was defined as an increase of greater than 5 in the percentage of antibodies resistant to elution by urea or an increase in the AI of more than 0.1, depending on the test used.

### Statistical analysis

Toxoplasma IgG avidity status was described as a continuous variable (% or AI specific value) and as a categorical variable (high, borderline or low, and stable vs. increasing avidity).

Continuous variables are presented as means with standard deviations or medians with quartiles and ranges when appropriate. Categorical variables are presented as numeric values and frequencies.

The groups were compared using bivariate analyses.

For all tests, *p-values* less than 0.05 were considered to indicate statistically significant differences. IBM SPSS statistics (version 20.01; IBM Corp., New York, NY, USA) was used to analyse the data and the figure was drawn in the R project for statistical computing, “R” (version 3.2.2. R Foundation for Statistical Computing,Vienna, Austria).

### Ethics

Our samples were collected from a project that was classified as a “quality insurance project” by the Regional Committee for Medicine and Health Research Ethics (2011/1310/REK.14.09.11). In addition, this study and the current publication was evaluated and approved by the Board of Patient Security at Oslo University Hospital (2012/9519.13.06.12). Because of the classification and long inclusion period, written consent was not obtained. Therefore patient information was de-identified prior to analysis, anonymised after analysis and will eventually be destroyed.

## Results

A total of 176 pregnant women with 542 serum samples were analysed (2–5 samples per woman, median 3 samples per woman). Samples from 109 (62%) women were analysed before 2005 and 67 (38%) after 2005.

All samples were positive for toxoplasma IgM. The mean maternal age at first serology was 29.9 years (SD 5.2 years, range 18–42 years). The patients’ demographic characteristics are described in Table 1.

**Table 1:** Characteristics of 176 Norwegian pregnant women undergoing amniocentesis on indication suspect Toxoplasma infection at Oslo University hospital, 1992–2013*.

Carachteristics	Number of women N	%
**Parity** (n = 169)		
P0	84	49.7
P≥1	85	50.3
**Nationality**		
Norwegian	142	80.7
Other	34	19.3
**County of residence**		
Oslo and Akershus	107	60.8
Nearlying counties**	41	23.3
Other***	28	15.9
**Education** (n = 121)		
9 year primary school	10	8.3
Secondary school	26	21.5
Higher education	85	70.2
**Antiparasitic treatment** (n = 166)	164	98.8

*Information missing on some of the parameters. N given in brackets

** Buskerud, Vestfold, Østfold, Hedemark

*** Telemark, Aust Agder, Vest Agder, Rogaland, Hordaland, Oppland

According to serologic group, the women were admitted for amniocentesis due to seroconversion (17, 9.7%), toxoplasma IgG antibody increase (31, 17.6%) and low toxoplasma IgG avidity and IgM positivity (128, 72.7%).

In 37 women (21%), avidity increased from low to high in less than three months, and in 139 women (79%), the avidity remained below the high threshold level for more than three months (Table 2).

**Table 2:** Development of toxoplasma IgG avidity in 176 Norwegian pregnant women according to serologic group and change in IgG avidity.

Serologic group at admittance	Total N	IgG-avidity increase form low to high	IgG-avidity increase within low/borderline range	No IgG-avidity increase	Fetal infection N	Fetal infection pr serologic group %
**IgG seroconversion** *	17	-	12	4	2	11.8
**IgG antibody increase** *	31	11	11	8	1	3.2
**Pos. IgM and low IgG-avidity**	128	26	39	62	1	0.8
**Total**	176	37(21.0%)	62(35.2%)	74(42.0%)	4	2.3

*one missing in the group below high limit

**Fig 1:**
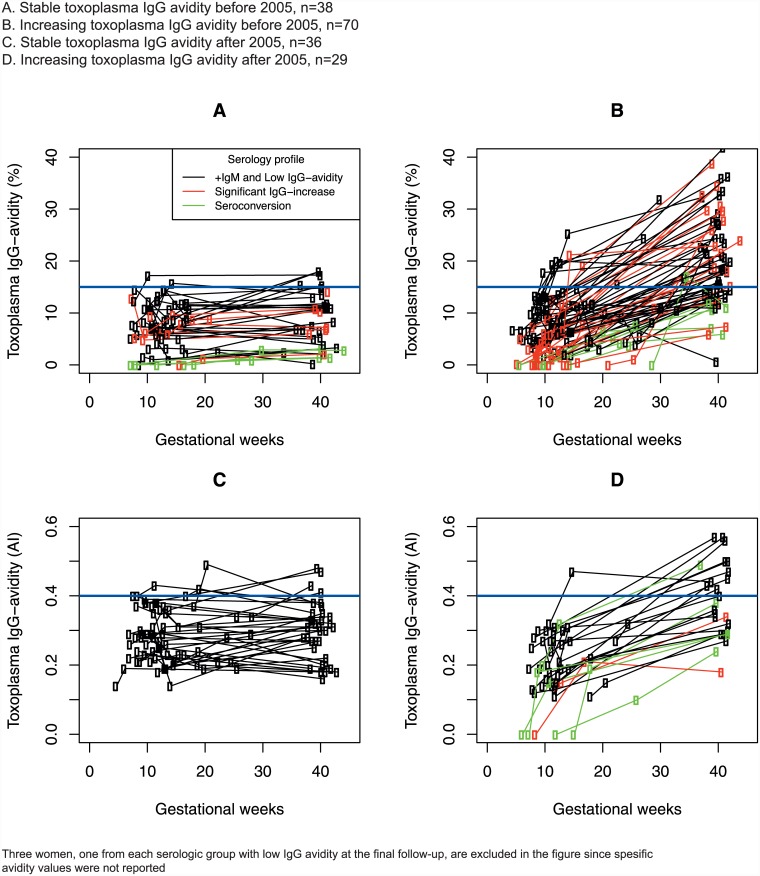
Toxoplasma IgG avidity changes during pregnancy in 176 Norwegian pregnant women undergoing amniocentesis at Oslo University Hospital, 1992–2013 on indication of suspect Toxoplasma gondii infection. A. Stable toxoplasma IgG avidity before 2005, n = 38 B. Increasing toxoplasma IgG avidity before 2005, n = 70 C. Stable toxoplasma IgG avidity after 2005, n = 36 D. Increasing toxoplasma IgG avidity after 2005, n = 29

However, in the latter group, the avidity increased significantly for 62 women (36%) without reaching the high level (Fig 1a–1d), and 74 women (43%) had stable low avidity during the observation period. For three patients, one from each serologic group, low avidity was reported at the final follow-up, but specific values were not reported; therefore, we do not know whether these three patients had stable or increasing avidity.

The median gestational age at the first toxoplasma antibody test during pregnancy was 10.6 gestational weeks (gw) (range 4.6–28.7 gw, quartile_25_ 8.4 gw, quartile_75_ 13.0 gw), and the median gestational age at the final test during pregnancy was 40.3 gw (range 27.3–44.3 gw, quartile_25_ 39.1 gw, quartile_75_ 41.1 gw).

For the 139 women with persistent IgG avidity below the high threshold, the median length of time between the first and final sample was 201 days (range 90–260 days, quartile_25_ 176 days, quartile_75_ 219 days). For two women, samples collected during the subsequent pregnancy were available (at 750 and 1836 days after the first sample) and still revealed low IgG avidity.

Amniotic fluid toxoplasma DNA PCR results were obtained after amniocentesis for all of the included women. In 171 (97.2%) of the children, toxoplasma serology results and toxoplasma DNA PCR results (obtained using cord blood, amniotic fluid and/or placenta tissue) were recorded at birth. Follow-up samples during the first year of life were obtained for 68 children (39.8%). Fetal infection was confirmed in four of the 176 children. For two of the infected infants, the mothers had seroconverted; for one infant, the mother had a significant IgG antibody increase, and for another infant, the mother had low IgG avidity and IgM positivity (Table 2). In the mothers with seroconversion, IgG avidity remained very low. In the mother with a significant IgG antibody increase, the IgG avidity increased from very low to high during the pregnancy, and in the mother with low avidity and IgM positivity, the IgG avidity increased but did not reach the high avidity threshold. There was no difference between women with increasing IgG avidity and women with stable avidity in regards to demographic characteristics, such as maternal age, nationality and parity. There was also no difference between these women regarding the duration of treatment (median 21 days, range 7–140 days, quartile_25_ 21 days, quartile_75_ 21 days) *(p = 0*.*23)*. Antiparasitic treatment was given to 98.8% of the women, at the median gestational day 103 (range 53–250 day, quartile_25_ 90 day, quartile_75_ 129 day). Median duration of treatment was 21 days (range 7–140 days, quartile_25_ 21days, quartile_75_ 21 days). Treatment was started before amniocentesis in 103 (62%) women, either azithromycin (88%) or spiramycin (12%). Pyrimethamine-sulfadiazine (alone or in combination with spiramycin) was given to13 women. Treatment was started prior to the second IgG avidity measurement in 32 (20%) women.

## Discussion

In 79% of the 176 pregnant women included in this study, toxoplasma IgG avidity remained below the high threshold for at least three months in pregnancy, with a median duration of low IgG avidity of approximately seven months. IgG avidity changed from low to high during the pregnancy in only 21% of the women. This finding is supported by previous studies [8, 10, 17].

In two of the women, low IgG avidity was also detected in the following pregnancy. This has been observed previously by other researchers and highlights the individual variability in the immune response to toxoplasma in regards to the IgG antigen binding force [17, 18]. In addition, the fact that 43% of the included women had stable IgG avidity throughout pregnancy and no fetal infection, also supports substantial individual variation in the immune response. These findings indicate that many of these women were most likely infected prior to conception. The kinetic of the serological response was not linear, but fluctuated in several women (Fig 1a–1d), and in one woman, the avidity even decreased throughout pregnancy (from 12.8% to 6.0%). Paired sera were not tested simultaneously; therefore, variation in IgG avidity may partly be explained by inter-assay variation.

The median duration of low avidity in our study was approximately seven months; however, this duration was likely to be longer as we did not follow all women until high avidity presented, and the point of acquisition was unknown for most women in this study [19]. Flori et al. found an average duration from the beginning of infection to reaching high IgG avidity of 14.5 months [10].

The interpretation of the IgG avidity results during pregnancy may be related to the type of assay used and the chosen cut-off value as no gold standard exists. Discrepant results between different tests are reported by both Lefevre-Pettazzoni et al., Murat et al. and Paul [9, 17, 20], but Petersen et al. found a good correlation between two toxoplasma IgG avidity methods [21]. Despite these discrepancies most researchers found an average duration of persistent low IgG avidity between five and 14 months in pregnant women [8, 10, 21]. Not surprisingly Flori et al. found shorter duration of persistent low IgG avidity when the avidity threshold in the Vidas test was lowered from 0.3 to 0.2 AI [10]. Therefore experience with the avidity assay and the cut-off value used must be emphasized in relation to recommending amniocentesis or not.

However, rather than to measure the exact duration of low IgG avidity, our aim was to estimate the frequency of a low avidity period of more than three months among randomly tested pregnant women. This information has important clinical implications for pregnant women tested in the first trimester and must be considered when planning prenatal diagnostics and treatment. The median time during pregnancy of first sampling in our cohort was approximately the eleventh gestational week. As previously mentioned, determining the time of infection at this stage in pregnancy is difficult. Considering our findings, there may be justification for performing an additional test three to four weeks after the confirmatory test (most often sampled three weeks after the initial test) in women with low avidity and IgM positivity before making a decision regarding the recommendation of amniocentesis. Amniocentesis should be recommended only if a significant increase in avidity is observed in addition to seroconversion or a significant antibody increase, as these collective findings clearly indicate an ongoing immunological process. In our study, no infected infants were found in the women with stable low IgG avidity and IgM positivity during pregnancy (other than in the women with seroconversion). Stable low IgG avidity in the first trimester most likely indicates preconceptional infection. However, this conclusion must be interpreted with caution due to the low number of infected offspring in our study.

Some researchers have found that antiparasitic treatment may alter the maturation of immunoglobulins, but others have found no correlation between immune status and treatment [8, 10, 19, 22, 23]. Alvarado-Esquivel et al. stated that treatment with pyrimethamine-sulfadiazine and atovaquone, but not spiramycin or azithromycin, impacted the antibody response in mice [24]. However, other researchers found a possible delay in IgG avidity maturation in spiramycin-treated pregnant patients [22, 23, 25]. We did not find any difference in the use or duration of antibiotic treatment between the groups with increasing versus stable avidity. However, most of our patients received antiparasitic treatment and our sample size was small, as in several previous studies. In our study, the most commonly used antibiotics were spiramycin and azithromycin with treatment duration of 21 days, initiated just before or right after amniocentesis. As amniocentesis was done around gw 16, most patients received antibiotic treatment between gw 15 and 18, and were therefore without treatment on average 20 weeks before the last toxoplasma IgG avidity test in pregnancy was performed. Only 13 women received pyrimethamine-sulfadiazine. Jenum et al. found no difference in IgG avidity between sera collected before and after treatment [8]. Sensini et al. found delayed maturation of IgG avidity the first four months after infection in a treated nonpregnant cohort compared to a nontreated group, but after seven months there was no difference between the groups [5]. The question remains, however, if the immunologic changes in pregnancy postpone the IgG avidity maturation as we have not retested the women after pregnancy.

We did not find any difference in maternal age between the high and low avidity groups, which is consistent with the findings of Lefre-Pettazzoni et al. [17].

The women were referred to the ultrasound unit at Oslo University Hospital for fetal examination due to possible primary *T*. *gondii* infection during pregnancy. Therefore, our study only describes the particular immune status of these pregnant patients, so our results should be applied with caution to the diagnosis of toxoplasma infection in the general population. However, our findings are consistent with the results of other antenatal studies [10, 19]. The mean maternal age in our sample did not differ from the mean age at delivery in the general population of our study area [15].

This study was retrospective and restricted to previously recorded data. Therefore, some information was incomplete, especially information concerning maternal health, education and parity. However, the laboratory records were thorough over the last twenty years, and maternal age and gestational week at blood sample collection were included in the laboratory records of all included patients.

In the last decade, a different method was used for IgG avidity analysis. However, the old and new tests use the same antigens and detergent for antibody-antigen binding. The main difference between the two methods is the avidity calculation method. An in-house validation of the comparability of results obtained using the two methods was performed before the method was changed, and no major differences were found between the methods. Nevertheless, the two methods for measuring IgG avidity may have resulted in slightly different cut-off thresholds, which may have affected the sensitivity and specificity of the test in regards to the exclusion and confirmation of recent toxoplasma infection. The Platelia IgG avidity test was used by Lachaud et al. and was found to have a sensitivity of 91.3% and a specificity of 98.5% [25].

## Conclusion

In more than 2/3 of the pregnant women in our study, toxoplasma IgG avidity remained low for three months or longer. Even if studies have generally found that IgG avidity matures over time, fluctuating avidity patterns make the accurate determination of primary infection difficult [26]. This emphasises the fact that high avidity can be used to exclude recent infection in first trimester but low avidity cannot verify acute infection; therefore, there is a diagnostic challenge associated with performing the first toxoplasma test as late as the third gestational month. Performing the test this late may lead to unnecessary diagnostic amniocentesis and treatment and unnecessary concern for the woman and her partner [26]. Therefore, the ideal timing for the first antenatal toxoplasma serology test is the very beginning of pregnancy.

In some patients, the final clinical decision can be improved by obtaining follow-up samples after approximately three to four weeks, before amniocentesis is performed. If IgG avidity remains low and stable in the first trimester, the probability that the infection occurred during the pregnancy is low, and the risk of fetal infection is even lower. However, if the avidity significantly increases, the woman should be advised to undergo prenatal diagnosis by amniocentesis.

Although the IgG avidity method has a limited ability to determine the onset of primary infection, there is no doubt that high IgG avidity helps to rule out infection in the pregnant women with persistent toxoplasma IgM positivity in the first four months of pregnancy.

## Abbreviations

gw: Gestational week
IgG: immunoglobulin G
IgM: immunoglobulin M
AI: avidity index
PCR: polymerase chain reaction
EIA: indirect enzyme-immunoassay
MEIA: microparticle enzyme-immunoassay
CMIA: chemiluminescence microparticle enzyme-immunoassay
